# A Comprehensive Approach to Medical Oxygen Ecosystem Building: An Implementation Case Study in Kenya, Rwanda, and Ethiopia

**DOI:** 10.9745/GHSP-D-21-00781

**Published:** 2022-12-21

**Authors:** Victoria Smith, Alana Changoor, Chloe McDonald, David Barash, Bernard Olayo, Steve Adudans, Tyler Nelson, Cheri Reynolds, Monica Cainer, James Stunkel

**Affiliations:** a Assist International, Ripon, CA, USA.; b University Health Network, Toronto, Canada.; c GE Foundation, Boston, MA, USA.; d Center for Public Health and Development, Nairobi, Kenya.; e Academy for Novel Channels in Health and Operations Research (ACANOVA Africa), Nairobi, Kenya.; f Health Systems Work, Inc., Kigali, Rwanda; Formerly of Health Builders, Kigali, Rwanda.

## Abstract

Medical oxygen is an essential treatment for life-threatening hypoxemic conditions and is commonly indicated for the clinical management of most leading causes of mortality in children aged younger than 5 years, obstetric complications at delivery, and surgical procedures. In resource-constrained settings, access to medical oxygen is unreliable due to cost, distance from production centers, undermaintained infrastructure, and a fragmented supply chain. To increase availability of medical oxygen in underserved communities, Assist International, the GE Foundation, Grand Challenges Canada, the Center for Public Health and Development (Kenya), Health Builders (Rwanda), and the National Ministries of Health and Regional Health Bureaus in Kenya, Rwanda, and Ethiopia partnered to implement a social enterprise model for the production and distribution of medical oxygen to hospitals at reduced cost. This model established pressure swing adsorption (PSA) plants at large referral hospitals and equipped them to serve as localized supply hubs to meet regional demand for medical oxygen while using revenues from cylinder distribution to subsidize ongoing costs. Since 2014, 4 PSA plants have successfully been established and sustained using a social enterprise model in Siaya, Kenya; Ruhengeri, Rwanda; and Amhara Region, Ethiopia. These plants have cumulatively delivered more than 209,708 cylinders of oxygen to a network of 183 health care facilities as of October 2022. In Ethiopia, this model costs an estimated US$7.34 per patient receiving medical oxygen over a 20-year time horizon. Altogether, this business model has enabled the sustainable provision of medical oxygen to communities with populations totaling more than 33 million people, including an estimated 5 million children aged younger than 5 years.

## Abstract (precis)

A social enterprise model was successfully implemented in Kenya, Rwanda, and Ethiopia that centers the production and distribution of medical oxygen at referral hospitals and equips them to act as supply hubs that help meet regional demand for affordable supply.

## Teaser

**Key Findings:**

- A social enterprise approach for medical oxygen production and distribution led to median increases in procurement of oxygen ranging from 112%–220% at customer hospitals, at an estimated cost of US$7.34 per patient treated.
- A key challenge faced by this model has been inconsistencies in cash flow, resulting from irregular purchasing patterns from hospital customers and infrequent schedules of reimbursement from the government. This was further exacerbated by national insurance schemes that do not sufficiently cover hospital costs.

**Key Implications:**

- This social enterprise model addresses oxygen scarcity in semirural and rural areas, where 60% of the population in sub-Saharan Africa resides. Pressure swing adsorption plant models offer a substantial advantage in mitigating transportation barriers that often preclude oxygen access to rural communities.
- To be successful, this model requires long-term budget commitments and prioritization of plant maintenance by funders, medical facilities, and implementers. Other enablers of success include investment in quality equipment and the early development of a long-term business strategy to achieve financial sustainability.

## INTRODUCTION

In 2017, the World Health Organization (WHO) categorized medical oxygen as an essential drug for the treatment of hypoxemia,^1^ with its utility spanning a diverse range of clinical disciplines including pediatrics, neonatology, obstetrics, surgery, and trauma.^2^ Ten to 15% of all pediatric hospital admissions require medical oxygen^3^, and the risk of death in hypoxemic children is 7 times higher than in other hospitalized children in some resource-constrained settings.^4^ Oxygen is also required for the emergency management of multiple obstetric conditions around the time of labor and delivery^5^ and is needed for 20% of hospitalized neonates.^3^^,^^6^^–^^8^ Moreover, oxygen is an essential component of safe surgery, and lack of access to safe surgery causes an estimated 16.9 million preventable deaths annually.^9^

While the coronavirus disease (COVID-19) pandemic illuminated the medical oxygen gap in resource-constrained settings, this gap is not new and was well documented before the pandemic.^10^^–^^14^ A study of 231 health care facilities across sub-Saharan Africa reported that less than 50% of facilities have consistent access to an oxygen source and 24.8% never have medical oxygen available.^15^ While efforts to comprehensively quantify the oxygen need gap are ongoing,^16^ a review of 64 high-care or intensive care units across 10 African countries found that 1 in 2 COVID-19 patients that died did not receive medical oxygen.^17^^,^^18^ Even as COVID-19 cases wane, there remains a clear need for long-term oxygen system strengthening as part of broader efforts to meet goals of universal health coverage and build resilience against future health shocks.

Even as COVID-19 cases wane, there remains a clear need for long-term oxygen delivery system strengthening as part of broader efforts to meet goals of universal health coverage and build resilience against future health shocks.

Reasons for oxygen scarcity are varied and complex. Across sub-Saharan Africa, for instance, the medical oxygen market is dominated by liquid oxygen (LOX) providers in capital cities.^19^ Because of the high capital costs to build a LOX plant, there are few LOX competitors, and some providers have exhibited monopolistic tendencies.^20^ As almost 6 in 10 sub-Saharan Africans reside in rural settings far from LOX plants,^21^^,^^22^ the cost and logistical barriers (e.g., transportation and lack of LOX infrastructure) make procuring oxygen from distant plants challenging for many facilities. The added cost and knowledge barriers associated with procuring and maintaining accessory equipment and devices, the absence of pulse oximeters, and a dearth of clinical training opportunities for health care workers increase the problem’s complexity. The cost to health facilities and patients, in addition to the previously described challenges, can present major hurdles to overcome. Therefore, while solutions to increase availability of the medical oxygen supply in resource-constrained settings are urgently needed, failure to address each barrier could compromise the success and sustainability of efforts to achieve lasting impact.

We describe a social enterprise approach in Kenya, Rwanda, and Ethiopia that was implemented (before the COVID-19 pandemic) to build an oxygen supply chain by establishing pressure swing adsorption (PSA) plants. We provide details of how the model was adapted to each context, an elaboration on achievements, enabling factors for success, and implementation barriers and bottlenecks. Whole life-cycle costing of the PSA model in each country is provided, along with a cost-per-patient analysis. We assess the program for its long-term sustainability, with considerations for replication and future research.

## USE CASES FOR MEDICAL OXYGEN SOURCES

There are 3 primary approaches to oxygen production and supply^23^: LOX, PSA plants, and oxygen concentrators. In most countries, a mix of oxygen sources can best serve the health system. Table 1 shares advantages and considerations for each oxygen source.

**TABLE 1.** Advantages and Considerations of Oxygen Sources

**Source**	**Advantages**	**Considerations**
LOX	Enormous production capacity (can supply countries and hospital systems).^19^^,^^22^ Maintenance responsibilities are often outsourced to businesses that are skilled in maintaining plants. Lower production costs than PSA plants.^19^^,^^24^ Can be stored in large tanks or cylinders, or piped directly to beds.^23^ Capable of providing high flow rates.^23^^,^^25^ Can be stored for use in unstable power situations.^19^^,^^23^	Plants are extremely expensive, creating high barriers to entry with one or few LOX supplier options.^19^ LOX plants typically located in central cities: transportation to very remote areas can be as expensive if not more so than the cost of the oxygen itself. Size of transportation vehicle or number of cylinders can also limit the amount of oxygen which can be transported.^19^^,^^26^ Recurring costs every time tanks or cylinders are refilled; tank or cylinder rental costs may also occur.^23^
PSA and VSA plants	Moderate production capacity (can supply a network of health care facilities). Can be located in more geographically diverse areas, meaning plants can be constructed on-site or near target hospitals, reducing transportation distance, costs.^19^^,^^23^^,^^24^ Can be stored in cylinders if cylinder filling capacity is included, or piped directly to beds.^19^^,^^23^ Cylinders can be used in unstable power situations.^19^^,^^23^ Capable of providing high flow rates.^23^	Requires regular maintenance, technical expertise, tools, test equipment, and spare parts to remain operational.^19^ Responsibilities and budget requirements often fall to the plant’s host hospital. Without them, the plant will fail.^19^^,^^23^^,^^24^ Requires reliable power supply, and a back-up generator with fuel is strongly recommended.^19^^,^^22^^–^^24^
Oxygen concentrators	Can provide a small, continuous supply of oxygen (can supply 1–2 patients at a time).^19^^,^^23^ Only recurring costs are power and maintenance.^19^ Very low capital barriers to entry, making it easier to acquire in resource-constrained settings.^27^	Very small production capacity. Flow rates not suitable for high flow patients.^19^^,^^23^ Dependent on a steady power supply; back-up power supply is strongly recommended, or back-up cylinders.^23^ Requires regular maintenance, technical expertise, tools, test equipment, and spare parts to remain operational; responsibilities and budget requirements often fall to the hospital. Without them, the concentrator will fail.^25^ Can be difficult for clinicians to identify when concentrators are only producing room air and not oxygen.

Abbreviations: LOX, liquid oxygen; PSA, pressure swing adsorption; VSA, vacuum swing adsorption.

### LOX

LOX is produced via a cryogenic air separation unit and distributed by large storage tanks and piping or cylinders. Good use cases for LOX include large hospitals located geographically close to a LOX facility, with local LOX storage and good transportation infrastructure. Advantages of LOX include large production volumes, as well as the outsourcing of complex maintenance requirements to a private business with expertise to ensure functionality. Transportation is typically its greatest disadvantage, as plants are usually installed in 1 or 2 urban locations nationwide. In areas with geographically dispersed populations, the cost of transporting oxygen can be a notable barrier.

### PSA Plants

PSA plants and vacuum swing adsorption plants both produce gaseous oxygen for distribution through piping or cylinders. PSA plants may be an appropriate oxygen source if a hospital is geographically separated from a LOX producer, or if supply routes are challenging or are often disrupted by storms, conflict, or natural disasters. Because PSA plants are smaller than LOX plants, they can be constructed on hospital grounds in multiple regions, thereby greatly decreasing transportation barriers. This is particularly helpful for semirural or rural communities, where 60% of the population in sub-Saharan Africa resides.^21^ A PSA plant is not recommended in hospitals that do not have a steady power source or the capacity to provide consistent maintenance.^19^^,^^22^^–^^24^

### Oxygen Concentrators

Portable oxygen concentrators produce gaseous oxygen on-site through individualized PSA technology, connecting directly to patients. With fewer recurring costs, oxygen concentrators can be a cost-effective supplement to other oxygen sources, or an option for small, remote health centers or wards that need small volumes and have a reliable power source. Like other oxygen sources, regular maintenance is required to ensure these devices are safe for patient use.^25^

## A SOCIAL ENTERPRISE APPROACH

In 2014, Assist International, a U.S.-based nongov-ernmental organization (NGO), collaborated with the Center for Public Health and Development (CPHD), a Kenyan-based nonprofit organization; Health Builders, a Rwandan-based nonprofit organization; and the GE Foundation to create a new oxygen delivery model to facilitate the generation, distribution, and clinical use of medical oxygen in public hospitals across East Africa. This model was initially implemented in partnership with Siaya County Referral Hospital in Kenya and Ruhengeri District Hospital in Rwanda. The model was then replicated in Ethiopia through the partnership of Assist International, the Amhara Regional Health Bureau, Dessie Referral Hospital, Felege Hiwot Referral Hospital, the GE Foundation, and Grand Challenges Canada. This program aimed to increase the regional availability of medical oxygen in rural and semirural locations, reduce the cost of oxygen to hospitals, and by doing so, increase the proportion of hypoxemic patients receiving oxygen therapy.

This program aimed to increase the regional availability of medical oxygen in rural and semirural locations and reduce the cost of oxygen to hospitals.

The social enterprise model varied slightly in each context. In Kenya, a traditional public-private partnership (PPP) was employed where the government hospital partnered with a for-profit social business enterprise (an enterprise whose mission is to improve a social aim while at the same time generating a profit). In Rwanda, the social enterprise was under the authority of a government hospital while remaining financially separate from the hospital. It was initially managed by an executive board with representation from the hospital, local government, and Health Builders; later, management responsibilities were transferred to hospital leadership. The social business enterprise model in Ethiopia took the form of a PPP comanaged by an international nongovernmental organization and the Amhara Regional Health Bureau, the local state chapter of the Ministry of Health (MOH) (Table 2).

**TABLE 2.** Summary of Social Enterprise Oxygen Models in Kenya, Rwanda, and Ethiopia

	**Siaya, Kenya**	**Ruhengeri, Rwanda**	**Bahir Dar, Ethiopia**	**Dessie, Ethiopia**
Business model	PPP managed by a for-profit social business enterprise	Social enterprise within a government hospital, managed by executive board	PPP comanaged by international NGO and Regional Health Bureau	PPP comanaged by international NGO and Regional Health Bureau
Time to break even, months	14	13	13	13
Percent change in quarterly procurement of 50 L oxygen cylinders at hubs (host hospitals where plants are located), %	12 (82 in Q1 to 92 in Q6)^a^	1403 (60 at baseline to 902 in Q8)	−27 (6,425 at baseline to 4,704 in Q8)^b^	73 (1,893 at baseline to 3,273 in Q6)
Health facilities served in first 24 months of operation, No.	39	9	60	39
Mean percent increase in procurement of 50 L oxygen cylinders in spoke customers (customers not hosting PSA plant on-site), %	112 (Q1 to Q6)	220 (baseline to Q8)	195 (at 24 months)	189 (at 24 months)

Abbreviations: NGO, nongovernmental organization; PPP, public-private partnership; PSA, pressure swing adsorption; Q, quarter.

^a^ Hub hospitals in Rwanda and Ethiopia had baseline figures for comparison. Unfortunately, these numbers were not available for Kenya. Because increases are calculated from Q1 instead of baseline, it is likely that figures here significantly underrepresent real changes. Data for Kenya was not available beyond Q6.

^b^ We deduce the decrease in oxygen consumed at this host hospital was due to the fact that total patient volume decreased at the host hospital, likely due to the opening of another teaching hospital in the same town. Staff members also reported that oxygen cylinders from the previous supplier were not often full when received, causing them to purchase more cylinders from other suppliers.

At the core of the approach was the strategy to center production and distribution of medical oxygen at large referral hospitals through PSA plants and equip them to act as cylinder “supply hubs” that help meet regional demand. Reduced-cost oxygen was either delivered to “spoke” hospitals or was procured from the nearby supply hub. Before this program, all the hub hospitals and most of the spoke hospitals purchased oxygen in cylinders at a premium from oxygen producers, typically LOX, hundreds of kilometers away.

Although financial details of the plants varied by location, grant funding provided by GE Foundation (for Kenya, Rwanda, and Ethiopia) and Grand Challenges Canada (for Ethiopia) was used to purchase oxygen plants, as well as provide funding for the social enterprise’s operational expenses until the plants could break even. In certain cases, grant funding from Grand Challenges Canada or GE Foundation was used to purchase additional equipment required for installation or operation of the oxygen plants.

Land and a building to house the plant mechanisms were donated by the hub hospital or the Regional Health Bureau, which retains ownership of the land and buildings, but loans the space in perpetuity to the oxygen plant. Electricity costs were covered by the local MOH or absorbed by the site hospital in exchange for free or discounted oxygen. In these cases, negotiations with the MOH resulted in an agreement to cover the cost of electricity through the MOH annual budget. The oxygen plants operated 24 hours per day, 7 days per week, except when minor power outages or maintenance occurred. Oxygen sales eventually covered the salaries of plant operators, along with regular maintenance costs, although a start-up period with grant funding was necessary before the plants were financially sustainable.

This model leveraged the key benefits of PSA plants—the ability to reduce transportation difficulties and costs by locating oxygen sources closer to regional hospitals—and blended it with one of the strongest benefits of the LOX model: outsourced maintenance. The highly technical and sometimes expensive maintenance required by the plant was outsourced to a social enterprise with a financial and missional interest in keeping the plants operational. Additionally, local technical expertise was strengthened as the social business enterprise employed, trained, and equipped plant technicians.

The social enterprise model leveraged the key benefits of PSA plants—the ability to reduce transportation difficulties and costs by locating oxygen sources closer to regional hospitals—and blended it with one of the strongest benefits of the LOX model: outsourced maintenance.

One of the greatest weaknesses and key risks of PSA plants is breakdowns. This can be mitigated with regular maintenance. However, in hospitals around the world, many PSA plants remain nonfunctional, as hospitals do not have the trained technicians, maintenance budget, spare parts, tools, or test equipment to keep plants operational. A map from the Every Breath Counts Coalition, a PPP supporting national governments to reduce pneumonia deaths in low- and middle-income countries, shows at least 165 PSA plants (as of October 2022) needing repair globally; 151 of these are located in sub-Saharan Africa.^28^ By focusing on regular maintenance, quality equipment, trained biomedical engineering teams, and financial sustainability from the program’s inception, the program avoided many of the pitfalls of other donated PSA plants.

In addition to increasing the availability of supply, the model was designed to provide solutions to several other barriers impeding oxygen access. This new ecosystem model used the following methods to achieve the program aims.

- Build oxygen plant infrastructure at host hospitals to reduce transport barriers
- Train a local team of plant technicians to provide sustained maintenance to avoid plant breakdowns and support long-term functionality
- Provide technical maintenance training to biomedical engineering teams on maintenance and repair of oxygen accessories and oxygen delivery systems
- Provide clinical training on safe and effective oxygen use
- Provide some accompanying oxygen accessories (e.g., cylinder regulators, flowmeters, pressure gauges), pulse oximeters, and consumables (e.g., cannula and oxygen masks in adult, pediatric, and neonatal sizes) to customer health facilities
- Create a sustainable supply system
- Transition ownership of the plants to in-country for-profit entities or government partners like public hospitals or regional health bureaus

To select a brand and model of PSA plant for this program, we conducted an informal survey of oxygen plants across Kenya and Rwanda. Surveyed plant technicians from the region suggested that AirSep plants, compared to other competitors, remained functional longest even when maintenance was neglected. The AirSep oxygen plants ASK (Kenya and Rwanda) and ASJ duplex (Ethiopia) can supply large volumes of medical oxygen for up to 20 years.^29^ This survey also suggested that Air Sep was more highly rated for its provision of local service. As service and equipment longevity would be key to program success, AirSep was selected.

Of note, when assessing the profitability of the program, the time to break-even analysis of the 3 case studies that follow does not include up-front capital costs (further breakdown in Whole Life-Cycle Costs section). Capital costs include the plant, generator, piping, the building surrounding the plant, shipping, initial maintenance training, and a small selection of spare parts. In this model, capital costs are grant funded, and operational costs are covered through the revenue generation model to keep costs low for hospitals. Many regional government health departments in resource-constrained settings have regularly funded budgets that can support ongoing oxygen plant operations with additional funds from the plant revenues but do not have operational budgets that allow for extensive up-front expenditure of purchasing PSA plants, infrastructure, and accessories. “Break even” is defined as the month when the earnings before interest, taxes, and amortization exceeded operational costs (staffing and costs of goods and services).

### Ethical Approval

Ethical approval for determining the number of patients receiving oxygen used in modeling the patient reach of the plant was provided by the Amhara Public Health Institute. Ethical approval for all other data was not required because no patient information was collected. No patients were involved in the design or implementation of this research.

## CASE STUDY 1: HEWA TELE, SIAYA, KENYA

**FIGURE 1:**
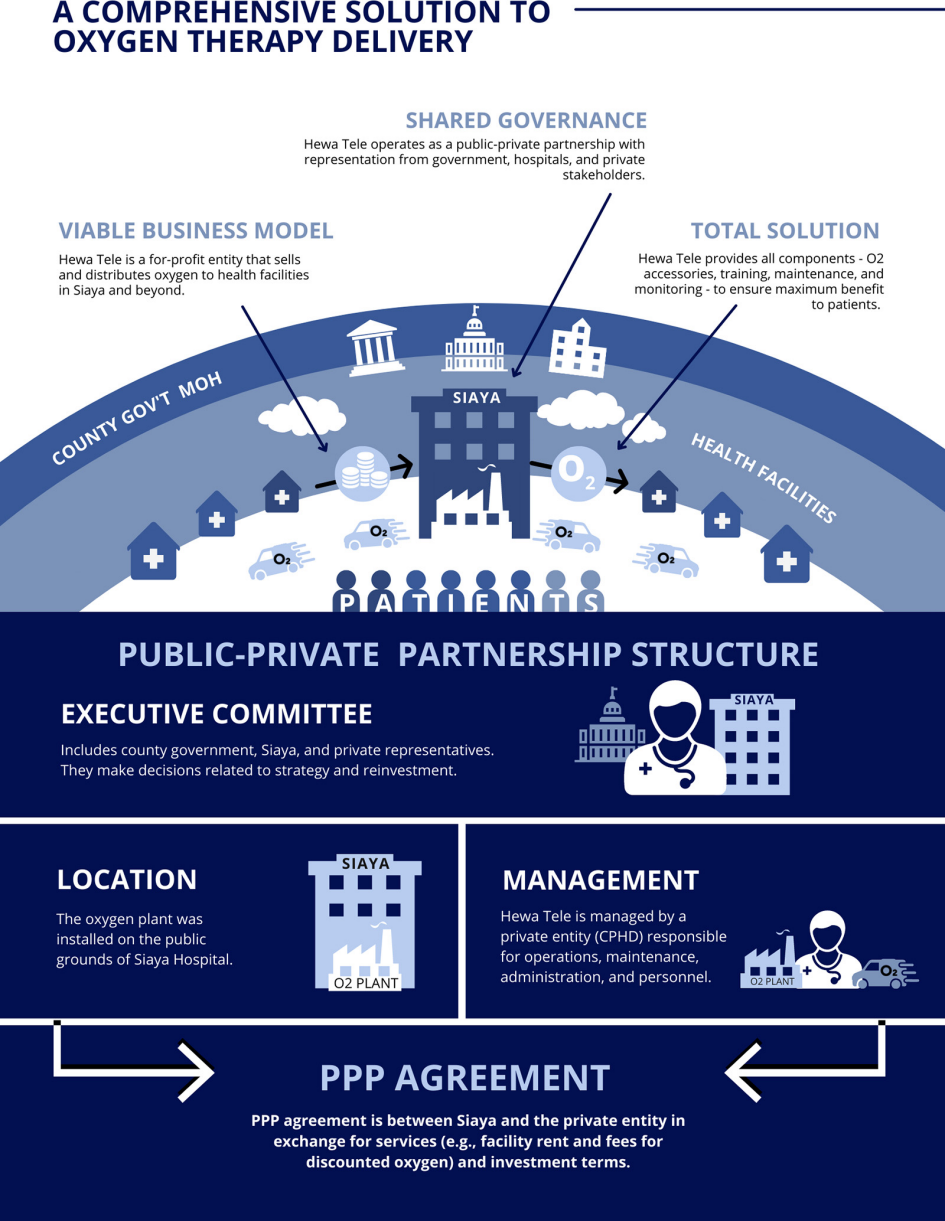
Hewa Tele Model of Comprehensive Oxygen Therapy Delivery in Kenya^a^ Abbreviations: CPHD, Center for Public Health and Development; gov’t, government; MOH, Ministry of Health; O2, oxygen; PPP, public-private partnership. ^a^ This oxygen model demonstrates a financially sustainable business model to improve oxygen availability by positioning Hewa Tele as a for-profit entity that sells and distributes oxygen to health facilities in Siaya and the surrounding areas. Hewa Tele provides a complete oxygen solution by providing all components required for oxygen delivery including clinical training, oxygen accessories, maintenance, and monitoring. Figure created by frog Consulting.

In 2014, Hewa Tele was developed as a private, for-profit subsidiary to CPHD in Siaya county, where there was a 90% gap between oxygen need and consumption rates, as estimated by a prelaunch market assessment performed by Hewa Tele. A social enterprise structure enabled Hewa Tele to sell and distribute medical oxygen cylinders under a sustainable, for-profit business model while keeping a central focus on improving the regional availability of supply for patients in need (Figure 1). Before the installation of the oxygen plant, most health facilities in the Siaya region purchased cylinders of oxygen from a LOX provider at a premium price (a 50 L cylinder sold for about US$57 in 2016, according to a market report from Hewa Tele).

A key breakthrough of the Hewa Tele model was a commitment from interdisciplinary stakeholders, funders, and governments through a PPP. Siaya County Referral Hospital (the “host hospital”) donated the site, along with reliable access to a power supply. Oxygen was sold in-hospital to the host hospital, as well as distributed to surrounding hospitals (Figure 1). GE Foundation funded the program and Assist International managed all procurements, logistics, and implementation. Hewa Tele supported plant operations and oxygen distribution, providing equipment maintenance and overseeing business processes including sales, services, and financial management. CPHD provided oversight of Hewa Tele and technical expertise around clinical training, including roll-out of training programs to accompany oxygen delivery for all levels of health care workers. Donated space for the plant and business operations minimized up-front costs, while strategic placement at the host hospital decreased the transportation burden and improved the supply, both at the host hospital and in surrounding counties. The private structure of the company allowed for flexibility with pricing, marketing, sales, and distribution.

A key breakthrough of the Hewa Tele model was a commitment from interdisciplinary stakeholders, funders, and governments through a PPP.

### Hewa Tele Model Outcomes

Business operations began breaking even monthly in February 2016, 14 months after opening. High-volume customers were essential to generate revenue, which allowed Hewa Tele to subsidize distribution costs to more remote facilities. Hewa Tele was able to reduce the cost of 50 L cylinders by 39% in comparison with previous oxygen suppliers. Spoke customers (customers without a PSA plant on-site) experienced an average 112% increase in oxygen use over 18 months (based on 8 hospitals), with some hospitals achieving increases of more than 200%. Oxygen use was measured by monitoring customer orders over time.

At the end of 2016, the program was transferred from Assist International program management to full Hewa Tele control. Facilities serving a catchment of 10 million people, and 1.5 million children aged younger than 5 years^30^^,^^31^ now receive regular oxygen delivery from Hewa Tele’s Siaya plant. The initial Siaya plant provides excess oxygen to other neighboring counties including Kisumu, Kakamega, and Bungoma. The model has since been replicated in 2 other locations in Kenya, Nairobi and Nakuru, where plants started operations in 2017. As of the last quarter of 2022, Hewa Tele’s network of plants serves more than 25 counties counties (Table 2).

## CASE STUDY 2: TOTAL OXYGEN SOLUTIONS, RUHENGERI, RWANDA

In 2014, Assist International collaborated with Health Builders to implement Total Oxygen Solutions, an oxygen program in Ruhengeri, Rwanda. Health Builders is a nongovernmental organization headquartered in Kigali that housed and supported Total Oxygen Solutions through expertise in public health systems, oxygen technology, and business model planning. Similar to the Hewa Tele model, the program in Rwanda was made possible through strong partnerships. The plant was established at a government facility, Ruhengeri District Hospital, with land donated through the Rwandan Ministry of Health for use by the program. Again, the GE Foundation funded the program, and Assist International managed all procurements, logistics, and implementation. Ruhengeri District Hospital is a tertiary government hospital with more than 400 beds serving a population of more than 350,000 people. As with many hospitals in resource-constrained settings, the consumption of oxygen, including at the host hospital, was far below the need before the installation of the oxygen plant. Before the plant installation, most hospitals trucked oxygen in cylinders from the capital city to all regions of the country. The oxygen plant operated as a social enterprise within a government health facility, governed by an executive committee with members from the local and central Rwandan government, Ruhengeri Hospital, and Health Builders. The plant was served by a dedicated power utility supply line, independent of the hospital’s power supply. Total Oxygen Solutions managed the plant, supported plant operations, provided equipment maintenance, and oversaw business processes including sales, services, and financial management. Funds from oxygen sales were designated for plant operations and ongoing maintenance costs.

### Total Oxygen Solutions Model Outcomes

To facilitate market entry and to reach customers at smaller, remote facilities, oxygen was strategically priced so that the cost of a cylinder included delivery. Like in Kenya, high-volume customers also helped subsidize the cost of delivery to smaller facilities. Deliveries usually occurred along a route, where oxygen for multiple hospitals or health centers was delivered from the same truck, also reducing delivery costs. Unlike Kenya, pricing was tiered, with closer facilities paying slightly less for the oxygen. In January 2016, the plant was generating sufficient revenue to consistently break even. Use of oxygen increased by more than 1400% (60 large cylinders per quarter at baseline to 902 large cylinders in quarter 4 of 2016) in Ruhengeri District Hospital 2 years after installation and by approximately 1800% after 5 years (1139 large cylinders in quarter 2 of 2019). The oxygen plant transitioned to Ruhengeri Hospital management in 2017, although finances of the plant remain separate from the hospital but under hospital management. As of the last quarter of 2019, Total Oxygen Solutions was providing regular oxygen cylinder deliveries for 13 health care facilities (some as far as 200 km away from the plant) serving a total catchment area of more than 3.1 million people (approximately 13.5% of the Rwandan population), including an estimated 428,000 children aged younger than 5 years.^30^

To enhance clinical capacity around oxygen use and equipment, Health Builders facilitated in-service training on oxygen therapy best practices for clinicians across 10 health care facilities in February 2016 (about 14 months after plant opening). An estimated 46 health care professionals have received training as of the last quarter of 2019. Data collected on oxygen consumption at 8 facilities pre- and postimplementation of the oxygen training program reported an average 53% increase in oxygen distributed in the quarter following clinical staff oxygen training compared to the quarter preceding the training, suggesting that staff training is an important parameter in appropriate increases in oxygen use use (Table 2).

To enhance clinical capacity, Health Builders facilitated in-service training on oxygen therapy best practices for clinicians across 10 health care facilities.

## CASE STUDY 3: THE ASSIST INTERNATIONAL MEDICAL OXYGEN PRODUCTION PLC, AMHARA, ETHIOPIA

After successful pilots of the oxygen program in Kenya and Rwanda, Assist International (and others) partnered with the Clinton Health Access Initiative to support the Ethiopian Federal MOH in developing the National Medical Oxygen and Pulse Oximetry Scale Up Road Map (Oxygen Road Map).^32^ In 2016, Ethiopia had 3 medical oxygen plants in operation and 1 additional plant under construction to serve the entire country, covering only 19% of the estimated need.^32^ To address this gap, the Federal MOH targeted 13 priority sites for new oxygen plants. Guided by the Oxygen Road Map, Assist International began working alongside the Federal MOH and the Amhara Regional Health Bureau in 2016 to increase the regional availability of oxygen in the Amhara Region. The region is the second largest in Ethiopia with a current population of more than 19 million people and had 3 of the 13 priority hospitals identified in the Oxygen Road Map, leading to its selection as the initial road map implementation location.

Implementation of the model in Ethiopia was guided by the same principles demonstrated in both previous oxygen programs: install an oxygen production plant closer to rural facilities based on need, train local teams to provide ongoing maintenance, train clinical hospital staff on best practices, keep the cost of oxygen low to health facilities, and use profits to reinvest in the sustainability of the business model. In 2018, Assist International Medical Oxygen Production PLC (public limited company) became a registered private enterprise operating in Ethiopia, and with the investment partnership of GE Foundation and Grand Challenges Canada, developed 2 demonstration oxygen plants at the Felege Hiwot and Dessie Referral Hospitals. The Amhara Regional Government approved legislation permitting the Regional Health Bureau to establish the Amhara National Regional State Medical Oxygen Production and Distribution Center (the Amhara Oxygen Center) which became the foundation for the PPP agreement between Assist International Medical Oxygen Production PLC and the Amhara Oxygen Center. Similar to previous models, the physical space and electricity for the plants were donated by the host hospitals, and each host hospital served as the first customer for the plant and social enterprise incubator. This system was a notable improvement compared to the existing oxygen procurement process in Ethiopia where most health facilities were required to retrieve oxygen in cylinders from central LOX hubs in Addis Ababa, often located hundreds of kilometers from their facilities. The mean price at baseline was about US$21 per 40L cylinder, with the mean transportation cost increasing these costs by an additional 74%.

### Assist International Medical Oxygen Production PLC Outcomes

The 2 plants provided more than 45,500 cylinders to 99 facilities in the first 19 months of operations, reducing the mean cost to hospitals by more than 50% even before transportation costs. Many public hospitals purchase oxygen from the plants with funds allocated from their yearly budget provided by the Amhara Regional Health Bureau.

The oxygen program in the Amhara Region has the potential to reach health care facilities with an estimated catchment population of 20.04 million people^33^ including an estimated 2.97 million children aged younger than 5 years.^34^ As with the partnerships put in place in Kenya and Rwanda, the long-term plans for the Amhara Region plants include the complete transition of plant ownership and management to the Amhara Regional Health Bureau after 5 years.

The oxygen program in the Amhara Region has the potential to reach health care facilities with an estimated catchment population of 20.04 million people.

Altogether the 4 plants have cumulatively distributed more than 209,708 cylinders of oxygen (as of October 2022) to 183 health care facilities and reach a combined catchment population of more than 33.1 million people, including an estimated 5 million children aged younger than 5 years. Table 2 provides a summary overview of the key parameters and outcomes of each implementation case study. Additional information on calculation methods is available in Supplement 1.

## WHOLE LIFE-CYCLE COSTS AND COST-PER-PATIENT ANALYSIS

PSA plant systems can produce substantial amounts of oxygen for 20 years or longer when properly maintained. Investors and funders interested in PSA systems can optimize a plant’s longevity and thereby maximize the number of patients that receive lifesaving medical oxygen over a plant’s lifetime by allocating appropriate resources towards maintenance and spare parts.

To help inform decision making around resource allocation and long-term budgeting, we prepared a whole life-cycle cost table of all capital expenditures and ongoing operational costs. We used real costs accrued as of September 2022 (in US$, Table 3) for each case study, an average of cost quotes for service contracts and spare parts packages, as well as valuation estimates for all costs contributed to the program in-kind (e.g., land and buildings by hospitals and electricity costs in some cases). Of note, some of the variations in costs between countries may be due to programmatic factors, such as sizing the investment to the needs of the community, the choice to install piping or not, as well as extraneous market factors and inflation. To project costs over a 20-year lifetime, we applied historic inflation rates from the year of installation to 2021. While the rate of inflation during the last 10 years averages to 2.04%, a projection of 2.5% per year was applied for future years to the U.S. dollar value of listed costs.

**TABLE 3.** Whole Life-Cycle Costs of Pressure Swing Absorption Plants in Kenya, Rwanda, and Ethiopia

	**Kenya, US$**	**Rwanda, US$**	**Ethiopia,**^a^ **US$**
Capital costs			
Plant, generator, and infrastructure Back-up generator Spare parts Surrounding infrastructure Electrical Installation labor Legal costs Shipping and taxes	319,813.59	269,393.68	438,749.95
Piping and manifold	218,016.66	92,000.00	0.00^b^
Cylinders, tools, and accessories Cylinders Cylinder accessories Oxygen plant toolkit Analyzers Pulse oximeters Shipping costs and taxes	213,706.29	134,250.77	107,605.87
Transportation Vehicles (e.g., trucks) Registration and insurance Inspection	53,814.60	38,498.00	53,485.41
Plant training	6,000.00	6,000.00	10,495.53
In-kind value of building/land	45,000	50,000	50,000
Total capital costs (excluding in-kind)	811,351.14	540,142.45	610,336.75
Total capital costs (including in-kind)	856,351.14	590,142.45	660,336.75
Yearly operating costs			
Plant labor	28,119.60	17,883.04	15,708.08
Accessories	5,839.04	1,215.80	23,813.32
Electricity	96,000.00	30,000.00	19,867.68
Fuel	3,830.56	2,827.40	10,250.60
Service contracts, labor	7,000.00	7,000.00	7,000.00
Service contracts, spare parts	17,000.00	17,000.00	17,000.00
Indirect/miscellaneous costs	14,873.96	5,085.00	8,892.20
Total yearly operating costs	172,663.16	81,011.24	102,531.88
Whole life-cycle cost over 20 years^c^	5,073,877.68	2,568,949.36	3,277,697.31

^a^ Ethiopia costs represent the cost per hub site.

^b^ Piping was not installed in Ethiopia.

^c^ Whole life-cycle cost incorporates year-over-year inflation.

**FIGURE 2:**
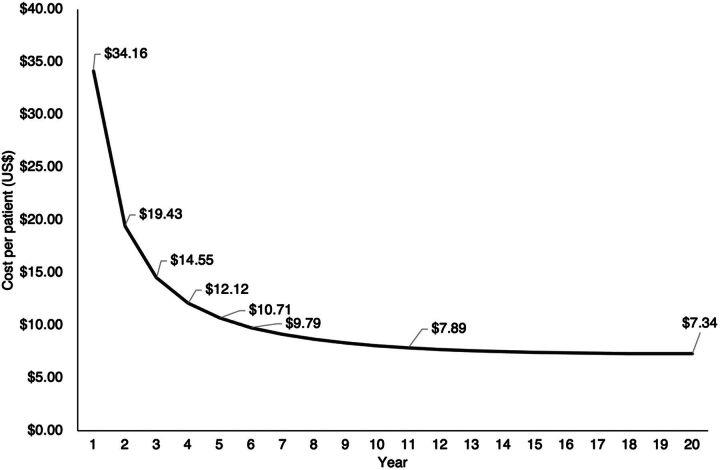
Cost of a Pressure Swing Adsorption Plant per Patient Treated With Medical Oxygen Over Time

A goal of this analysis is to clarify the potential patient reach and cost trajectory of a well-sustained PSA plant. To achieve this, in Ethiopia, an estimation of whole life-cycle cost per patient is modeled using data from a sample of hospitals regularly purchasing oxygen. Supplement 2 provides more details on the modeling approach. Overall, an estimated 22,333 patients are modeled to directly receive medical oxygen treatment in Ethiopia, per plant, per year of operations. With an assumed life span of 20 years, this equates to 446,660 patients and a US$7.34 cost per patient receiving medical oxygen therapy. This analysis demonstrates that with adequate provisions for the maintenance of a plant, there is a steep decrease in the cost-per-patient receiving treatment over time. As illustrated in Figure 2, there is a sharp cost decrease from US$34.16 per patient treated in year 1 of operations to less than US$10 per patient treated within 6 years of operations, which plateaus to about US$7.34 per patient treated after 20 years of operations. These cost-per-patient estimates notably include the value of in-kind donations of buildings and electricity, for purposes of generalizability across different models of investment.

While there is a dearth of academic literature on the impact and operational feasibility of PSA oxygen plants, in the team’s programmatic experience it is common to find instances where PSA plant systems have been purchased without the requisite budget or allocation of resources toward long-term maintenance, undercutting the longevity of plant functionality. In other cases, plants may be installed without investment in training local technicians, with plants left unmonitored and poorly maintained after an initial warranty period.

The findings of this analysis underscore the importance of functional status and plant maintenance, as poorly maintained plants are likely to have a shorter life span. Examples of scenarios that could compromise plant functionality include breakdowns in the booster compressor (equipment used to fill cylinders) or a failure of the air compressor, affecting the purity and delivery of adequate oxygen. These are examples of challenges that can be addressed through regular preventative and corrective maintenance by a trained team of technicians.

The findings of this analysis underscore the importance of functional status and plant maintenance, as poorly maintained plants are likely to have a shorter life span.

This finding raises important considerations for global health actors interested in addressing the persisting inequities in medical oxygen access globally. To date, literature on the cost-effectiveness of oxygen systems has focused exclusively on concentrator-based systems. A 2021 systematic review and meta-analysis of 4 concentrator-based programs estimated an average cost-effectiveness ratio of US$62 per disability-adjusted life year averted (range: US$44–$225).^35^ A more recent study on a solar-powered oxygen program estimated costs of US$26 per patient treated and US$542 per life saved.^36^ These existing estimates provide strong evidence that concentrator systems are cost-effective health interventions. However, while concentrators are well suited to improving oxygen availability in small, rural health care facilities,^36^^–^^40^ they are not a standalone solution to larger health care facilities, and with most models able to deliver flow rates of 10 L/minute or less, they may not be able to offer the higher levels of flow needed to effectively treat some hypoxemic patients. To the team’s knowledge, this cost-per-patient data is the first available for a large-scale PSA oxygen system. While estimates of cost per disability-adjusted life year averted or life saved were beyond the scope of this program, these are intended directions for further evaluation.

## LESSONS LEARNED

### Enablers of Success

PSA plants require a large up-front capital investment. To fully realize the potential of this investment, long-term vision is required. It is this team’s experience that the procurement of a plant must be accompanied by adequate due diligence and a long-term budget to support maintenance needs and ongoing operational costs. If plants lack the resources to regularly perform preventative and corrective maintenance, lack the resources to procure spare parts, and/or lack technicians with the requisite expertise, the longevity and functionality of the plant will be compromised.

The procurement of a PSA plant must be accompanied by adequate due diligence and a long-term budget to support maintenance needs and ongoing operational costs.

From the beginning of the program, we made provisions to ensure long-term sustainability was prioritized. This included conducting due diligence of different brands with the selection of plants that had a demonstrated track record of robustness and quality functional performance. Maintenance has remained a top priority. The installation of plants was accompanied by comprehensive technical trainings, as well as a budget for service contracts, technical teams, ongoing trainings, back-up infrastructure, and spare parts. Finally, we also prioritized financial sustainability from the program’s inception to make the model self-sustaining beyond the timelines of donor funding. Developing tailored business strategies and the ongoing iteration of these strategies has been instrumental, with plants now able to fund their own ongoing operational expenses through generated revenues.

### Implementation Challenges

At implementation, maintaining a consistent cash flow was difficult due to irregular hospital purchasing and infrequent reimbursement for oxygen by local governments. While increasing numbers of hospitals that purchased from these plants over time, the oxygen volumes purchased by each customer were sometimes unpredictable. The irregularity of purchase patterns among customers made it difficult to accurately estimate monthly revenue. Additionally, infrequent reimbursements from regional health authorities slowed payment for oxygen by customer hospitals. For these reasons, maintaining a steady cash flow for business expenses could be difficult, even when the business was selling high volumes of oxygen cylinders.

Additionally, national health insurance schemes often do not adequately provide for the cost of purchasing oxygen, passing substantial costs onto hospitals. In most countries, an oxygen budget is provided to appropriate public health facilities through the ministry of health. The way the oxygen budget is distributed varies. Often, the budget does not provide payment for the full cost of oxygen at the health facility. For example, the Rwandan government’s insurance plan covered oxygen therapy, but it only provided a flat, one-time payment. This meant that if patients needed more oxygen than was covered, the hospital would provide it at a loss. This was a key financial disincentive to provide the appropriate amount of oxygen therapy. In Ethiopia, many hospitals requested additional guidance from regional health leadership to know whether or not patients should be charged to make up oxygen deficits. This occurred even with prices to hospitals 50% lower than preprogram rates in some locations. In some countries, hospitals generally determine the price of oxygen to patients, which can influence patient access to oxygen therapy.

### Best Practices

The program team iterated upon the implementation approach over time to incorporate measures that address hospital-related bottlenecks to oxygen access. Close engagement with recipient hospitals elucidated clear gaps in the available inventory of supporting equipment and supplies. This is important because even when the local supply of oxygen has increased, broken or inadequate stock of key equipment and supplies like oxygen cylinders, consumables (size-specific cannulas and face masks, especially neonatal), and accessories (e.g., regulators, flowmeters, and oximeters) contributed to unused oxygen. Solving these barriers is key to providing oxygen to patients in need.

The program team iterated upon the implementation approach over time to incorporate measures that address hospital-related bottlenecks to oxygen access.

Similar limitations can result from gaps in clinical capacity. Implementing clinical training opportunities resulted in considerable synergies and resulted in large increases in oxygen usage as well as reductions in oxygen wastage. For instance, when clinicians are better equipped to identify patients that are hypoxemic earlier or are trained on the appropriate titration of medical oxygen, there is more likely to be a health impact associated with improved supply. These examples highlight the value-add and importance of considering the entire oxygen ecosystem and align with the findings and conclusions of other organizations implementing oxygen system strengthening solutions.^12^^,^^36^^–^^41^

### Future Considerations

Although the key achievements and operational sustainability are positive indications of success, as with all models, some limitations should be considered before replicating this program elsewhere. The model requires considerable capital investment for procurement of the oxygen plant, cylinders, training, and related materials and sufficient cash flow to support initial operations, including staff salaries, before revenue generation and break-even cash flow. As oxygen availability increases in health care facilities, the cost can be passed down to the patient. This varies widely based on the setting but is worth noting as this has important implications and emphasizes the need to keep customer costs low. To ensure continued plant operations, ongoing plant maintenance must be prioritized. Across all oxygen implementation models, equipment maintenance is often overlooked. As a result, this model invested heavily in maintenance training and support. This investment must be upheld to ensure sustainability of both the business model and improvements in oxygen availability. Procuring any medical oxygen equipment without a long-term maintenance plan will undermine the potential of the initial investment and should not be expected to yield sustainable results. Future research should investigate the operational feasibility and sustainability of other oxygen system strengthening approaches, including alternative models of maintenance and revenue generation.

## CONCLUSION

To the team’s best knowledge, this is the first published evaluation of a PSA-based oxygen system strengthening solution. We deployed a social business enterprise strategy to promote long-term sustainability and create a localized supply for rural hospitals struggling with oxygen scarcity. The break-even point at each plant was achieved within 14 months with considerable increases in oxygen procurement at both host and spoke hospitals.

Since 2013, Assist International and colleagues at CPHD and Health Builders have partnered with GE Foundation, Grand Challenges Canada, and national and regional government partners in Kenya, Rwanda, and Ethiopia to demonstrate that oxygen plants operated as a social enterprise can be implemented across distinct geographies and adapted to local environments. All case studies illustrate that businesses can profitably support supply chain management of medical oxygen at reduced costs for hospitals over the long term. This implicates PSA-based social enterprise models as a viable and sustainable strategy for changing the landscape of oxygen access for patients in need.

## Supplementary Material

GHSP-D-21-00781-supplements.pdf

## Notes

**Peer Reviewed**

**First published online:** November 22, 2022.
