# Supplementary material for: A Comprehensive Approach to Medical Oxygen Ecosystem Building: An Implementation Case Study in Kenya, Rwanda, and Ethiopia
Source: Glob Health Sci Pract. 2022 Dec 21;10(6):e2100781. doi: 10.9745/GHSP-D-21-00781 (PMC9771461; doi:10.9745/GHSP-D-21-00781)
Supplement: GHSP-D-21-00781-supplements.pdf [file GHSP-D-21-00781-supplements.pdf]

Supplement to: Smith V, Changoor A, McDonald C, et al. A comprehensive approach to medical oxygen ecosystem building: an implementation case study in Kenya, Rwanda, and Ethiopia. *Glob Health Sci Pract*. 2022;10(6):e2100781. https://doi.org/10.9745/GHSP-D-21-00781

SUPPLEMENT 1. Methods for Monitoring Oxygen Procurement

This supplement provides an elaboration on methods for calculations presented in Table 3 on procurement of oxygen cylinders across hub and spoke hospitals.

Data Sources

1. Monthly data collection: Program records on the specific volumes of oxygen distributed to each host and spoke customer hospital were collected. The number of spoke customer hospitals purchasing from the plant were inventoried so that an analysis of how many times they procured oxygen from the plant over a given period of time could be tracked.

2. Monthly expense tracking: Revenues gained and costs accrued by the plant were tracked monthly.

Calculations:

3. “Break-even”: In each case, the time to “break even” was calculated by counting the number of months until the social business enterprise was able to pay operating costs (power, staffing, oxygen accessories, marketing, transportation, and fuel.) This occurred when EBITA (earnings before interest, taxes, and amortization) was greater than zero. “Capital costs” outlined in Table 2 were donated to the program and were not included in the “break even” analysis.

4. “Percent change in oxygen procurement”: Percentage change in the procurement of 50L oxygen cylinders at hubs (host hospitals where plants are located) and spokes (customers not hosting PSA plant on site) were calculated by converting monthly volumes of oxygen procured into 50L cylinder units. (Hospitals use several different cylinder sizes. 50L cylinder size was chosen for comparability across programs.) Months were aggregated into quarters, and percent change was calculated using the formula $[(V2-V1)/|V1|]\times 100=?$

a. Hubs: Baseline data for the quarter prior to the oxygen plant opening was compared to the last available quarter of data for each plant.

b. Spokes: Data for program Q1 was compared to the last available quarter of data for each plant.

Of note, baseline figures before plant installation were not available for Kenyan hospitals. Figures for Kenya have been calculated using Q1 of program data. As hospitals typically began to increase oxygen procurement immediately after the plants became operational, it is likely all Kenya figures underrepresent real increases.

Supplement to: Smith V, Changoor A, McDonald C, et al. A comprehensive approach to medical oxygen ecosystem building: an implementation case study in Kenya, Rwanda, and Ethiopia. *Glob Health Sci Pract*. 2022;10(6):e2100781. https://doi.org/10.9745/GHSP-D-21-00781

SUPPLEMENT 2. Modeling Patient Reach of Ethiopian PSA Plants

As part of the Ethiopia program's ongoing Monitoring and Evaluation, several data points have been collected from a sample of 14 "program hospitals", defined as such because they are part of broader and ongoing oxygen-system evaluations. These include the 2 referral (high-volume) hub hospitals, 2 general (medium-volume) spoke hospitals, and 10 primary (low-volume) spoke hospitals. The data from these hospitals were used to create a model to estimate the plant's total patient reach. These are described below and used in this study to derive cost-per-patient estimates.

<u>Modeling Approach, Data Sources & Assumptions:</u>

1. Total Daily Patients Receiving Oxygen: Data collectors visited surgical, obstetric, emergency, pediatric, ICU, and neonatal wards in the morning, afternoon, and evening of each program hospital (n=14) over a period of 20 days. In these wards, collectors directly observed the number of new and unique patients on oxygen during each ward visit. Ethical approval for this was granted by the Amhara Public Health Institute. All patient data was deidentified upon collection.

2. These estimates of total patients receiving oxygen were averaged across hospital type. While the plants were serving 100% of the oxygen needs of their host referral hospitals, each plant contributed only a percentage of total oxygen procured by other hospital customers. Team members heavily engaged in routine monitoring and evaluation of oxygen usage across spoke hospital sites informed the following assumptions: of the 12 program spoke hospitals, an estimated 70% of oxygen needs are met through the newly installed plants as compared to ~40% of other non-program hospital consumers.

3. An inventory of the total number of months in the year 2020 that each recorded hospital consumer procured oxygen from the program plants was tallied and converted to days. Estimates of total daily volume of patients on oxygen were extrapolated using the 14-hospital sample and discounted by the assumptive percentages of oxygen needs met by the program PSA plant versus other sources. The total across all hospitals was then summed.

4. To account for increases in the percentage capacity at which the plants were being operated, plant operators provided the monthly operating capacity of the Ethiopia plants. As the operating capacity increased steadily over time, an average of the operating capacity across all months in 2020 was tabulated. Using ratio formulas, the total number of patients estimated to receive oxygen in step #3, was divided by the average % capacity and multiplied by 90%. This resulted in an estimate of 22,333 patients receiving medical oxygen sourced from a program plant, operating at 90% capacity, per year.

5. This was multiplied out by 20 years (projected lifespan of a well-maintained PSA plant), to arrive at a total estimate of 446,660 patients receiving oxygen from a PSA plant over its entire lifetime.
